# Production and Characterization of a Bioflocculant from *Pichia kudriavzevii* MH545928.1 and Its Application in Wastewater Treatment

**DOI:** 10.3390/ijerph19063148

**Published:** 2022-03-08

**Authors:** Phakamani H. Tsilo, Albertus K. Basson, Zuzingcebo G. Ntombela, Tsolanku S. Maliehe, V.S.R. Rajasekhar Pullabhotla

**Affiliations:** 1 Department of Biochemistry and Microbiology, Faculty of Science, Agriculture and Engineering, University of Zululand, P/Bag X1001, KwaDlangezwa 3886, South Africa; ntombelaz@unizulu.ac.za (Z.G.N.); bassona@unizulu.ac.za (A.K.B.); sidttmaliehe@gmail.com (T.S.M.); 2 Department of Chemistry, Faculty of Sciences, Agriculture and Engineering, University of Zululand, P/Bag X1001, KwaDlangezwa 3886, South Africa

**Keywords:** *Pichia kudriavzevii* MH545928.1, bioflocculant, removal efficiency, wastewater

## Abstract

A variety of flocculants have been used to aggregate colloidal substances. However, recently, owing to the adverse effects and high costs of conventional flocculants, natural flocculants such as microbial flocculants are gaining attention. The aim of the study was to produce and characterize a bioflocculant from *Pichia kudriavzevii* MH545928.1 and apply it in wastewater treatment. A mixture of butanol and chloroform (5:2 *v*/*v*) was used to extract the bioflocculant. Phenol–sulphuric acid, Bradford and Carbazole assays were utilized for the identification of carbohydrates, proteins and uronic acid, respectively. Scanning electron microscopy (SEM) and elemental detector were employed to determine the surface morphology and elemental compositions. The removal efficiencies were 73%, 49% and 47% for BOD, COD and P, respectively. The bioflocculant (2.836 g/L) obtained showed the presence of carbohydrates (69%), protein (11%) and uronic acid (16%). The bioflocculant displayed a cumulus-like structure and the elemental composition of C (16.92%), N (1.03%), O (43:76%), Na (0.18%), Mg (0.40%), Al (0.80%), P (14.44%), S (1.48%), Cl (0.31%), K (0.34%) and Ca (20.35). It showed the removal efficiencies of 43% (COD), 64% (BOD), 73% (P) and 50% (N) in coal mine wastewater. This bioflocculant is potentially viable to be used in wastewater treatment.

## 1. Introduction

Recent industrialization and urbanization have led to high rates of water pollution and water scarcity [1]. Major sources of water pollution are untreated, toxic, domestic, industrial effluents and agricultural byproducts. Pollutants such as dyes found in wastewater are often detrimental to humans, animals and aquatic life. A number of physico-chemical treatment methods, such as chemical precipitation, ion exchange, activated carbon adsorption and reversed osmosis, have been used for the removal of these pollutants. However, some of these treatment methods are expensive and impose environmental and human threats [2]. Thus, innovative, cheap methods such as flocculation, which are effective, easily sustainable and economically viable, are methods of choice.

Flocculation is a purification process whereby destabilized colloids agglomerate to form flocs [3]. It is facilitated by the reduction in repulsion forces through destabilization of colloidal particles and lowering of the energy barrier between them, thus enhancing their aggregation [4,5]. Thus, floc formation is described by the coagulation theory, whereby the likelihood of particles to bind together depends on the possibility of their collision and on the probability of adhesion following their collision [6,7]. The strength, density and size of flocs greatly depend on the interparticle bonds within the constituents, colloid size, suspended sediment concentration, mineralogy and fluid shear [7]. The strength of the flocs depends on interparticle bonds within the constituents of the aggregates [8]. The flocs can be easily removed by sedimentation and filtration processes. However, the characteristics of flocs play a vital role in the subsequent effective processes of settling and filtration [9].

Flocculants can be grouped according to their chemical constituents or make-up. The three main groups of flocculants are: (a) inorganic, (b) organic synthetic and (c) natural occurring flocculants [10]. Inorganic flocculants (aluminum chloride (Al^3+^) and ferric chloride (Fe^3+^)) are used predominantly in wastewater treatment because of their cost-effectiveness [11]. However, they have inherent usage limitations due to the fact that large amounts are required and are highly sensitive to pH variations and low temperatures [12]. Organic synthetic flocculants such as polyacrylic acid, polydiallylmethyl ammonium chloride and polyacrylamide were also used as alternatives [13]. This is because they have high efficiency, are convenient to utilize, are highly soluble in aqueous solutions and are not affected by pH changes. However, their monomers have high resistance to biodegradability, thereby instituting environmental pollution. Moreover, most of them are produced from petroleum-based products, which have a high margin of detrimental effects on humans and the environment [14]. Thus, the drawbacks of inorganic and organic flocculants necessitate more research into natural occurring flocculants such as bioflocculants (microbial flocculants) [15].

Bioflocculants are biopolymers of microorganisms produced during their growth phase. Complex multi-chain, hefty molecular polymers containing repeating branched polyols, sugar derivatives, proteins and glycoproteins make up the bioflocculants [16]. Bioflocculants can be produced at high rates depending on the culture parameters and on medium composition. They are effective [17], inert to pH fluctuations [18], nontoxic in nature [19], harmless to humans and animals [15] and lack secondary pollution [20]. However, the usage of bioflocculants is due to low production yields by microorganisms [21]. Moreover, the bioflocculation characteristics and mechanisms of action are still not fully understood in comparison to conventional flocculants [22]. This is because the characteristics of bioflocculants differ between bioflocculant producers and often tend to result in diverse mechanisms [23]. 

In our previous study, we isolated, identified and optimized culture conditions for a bioflocculant producing *Pichia kudriavzevii* MH545928.1 from Kombucha tea SCOBY [24]. *Pichia kudriavzevii* is the teleomorph of the *Candida krusei* that was previously regarded as *Issatchenkia orientali*. This fungus is found abundant in the environment, especially in soil, fruits and fermented beverages. It is mainly utilized in the fermentation of alcoholic beverages [25,26]. However, to our knowledge, this fungus has not been studied as a bioflocculant-producer. The aim of this study was to extract a bioflocculant from *P. kudriavzevii* MH545928.1 by using the solvent extraction method, characterize it by different standard methods and use it to treat coal mine wastewater. Moreover, the dye removal efficiency of the bioflocculant was also evaluated.

## 2. Materials and Methods

### 2.1. Chemicals and Production Medium

The reagents, chemicals and media used were procured from Sigma-Aldrich (St Louis, MO, USA). The medium for bioflocculant production composed of glucose (20.0 g), KH_2_PO_4_ (2.0 g), K_2_HPO_4_ (5.0 g), (NH4)_2_SO_4_ (0.2 g), NaCl (0.1 g), CH_4_N_2_O (0.5 g), MgSO_4_ (0.2 g) and peptone (0.5 g) in a Liter of the autoclaved Kombucha tea. 

### 2.2. Source of Fungus

*P. kudriavzevii* MH545928.1 was previously isolated from Kombucha tea with a SCOBY, which is an acronym for Symbiotic Culture of Bacteria and Yeast, which was purchased from Greenheart Organics Pinetown in Durban KwaZulu-Natal Province, South Africa. It was identified by internal transcribed spacer (ITS) rRNA gene sequence analysis and stored at −80 °C in 50% glycerol in the Department of Biochemistry and Microbiology laboratory at the University of Zululand, South Africa. Prior to being used, *P. kudriavzevii* MH545928.1 was resuscitated on potato dextrose agar (PDA).

### 2.3. Extraction and Purification of the Bioflocculant

The method by Okaiyeto et al. [2] was adopted in order to extract and purify a bioflocculant from *P. kudriavzevii* MH545928.1. The fungus that was resuscitated on PDA was inoculated into production medium (glucose (20.0 g), KH_2_PO_4_ (2.0 g), K_2_HPO_4_ (5.0 g), (NH_4_)_2_SO_4_ (0.2 g), NaCl (0.1 g), CH_4_N_2_O (0.5 g), MgSO_4_ (0.2 g) and peptone (0.5 g) in a liter of the autoclaved Kombucha tea). It was cultured at 35 °C, pH 7 and 140 rpm for 60 h. After 60 h of fermentation, the broth was centrifuged at 8000 rpm for 15 min at 4 °C to remove bacterial cells. About one volume (1000 mL) of distilled water was added into the cell-free supernatant, mixed properly and re-centrifuged at 8000 rpm for 15 min at 4 °C. About 2 volumes (2000 mL) of ice-cold ethanol were added into the supernatant, mixed properly and stored for 12 h at 4 °C. In order to obtain crude bioflocculant, the precipitate was vacuum dried. The crude bioflocculant was then re-dissolved in distilled water (100 mL) to form a solution (*w*/*v*). Butanol (n-butyl alcohol) and chloroform (5:2 *v*/*v*) (100 mL) mixtures were added. The mixture was thoroughly shaken and left at room temperature to stand for 12 h. Thereafter, the precipitate was centrifuged at 8000 rpm for 15 min and vacuum-dried to obtain a purified bioflocculant. The weight of the dried bioflocculant was measured and expressed in g/L. 

### 2.4. Solubility Assay of the Purified Bioflocculant 

Solubility assay of the purified bioflocculant was determined visually using a shake-flask method, according to Zaki et al. [27]. The bioflocculant (0.05 g) was dissolved in 2 mL of different solvents (water, chloroform, ethanol, methanol and butanol), agitated and allowed to stand for 2 min. Thereafter, the solubility of the extract was determined.

### 2.5. Physicochemical Analysis of the Purified Bioflocculant

#### 2.5.1. Chemical Composition Analysis

The phenol–sulfuric acid method was employed in order to determine the total sugar content, and D-glucose was utilized to prepare the standard curve [28]. Briefly, 0.2 g of the purified bioflocculant was poured into a beaker containing 100 mL of autoclaved distilled water. About 0.2 mL of phenol was pipetted into the solution together with 1.0 mL of sulfuric acid. The mixture was allowed to stand at room temperature for 10 min, followed by vigorous agitation for a minute. Thereafter, absorbance was measured at an optical density of 490 nm using a spectrophotometer (Unic-7230, Shanghai Lianhua Company, Shanghai, China). Bradford assay with bovine serum albumin (BSA) as standard was used to measure the total protein content of the produced bioflocculant [29]. Briefly, 20 µL of each solution was pipetted into a 96-well plate. Bradford reagent (180 µL) was poured into dilutions. The mixture was left to stand at room temperature for 2 min. Thereafter, the solutions were read at 595 nm using a spectrophotometer (Unic-7230, Shanghai Lianhua Company, Shanghai, China). The colorimetric method using carbazole reagent was used in determining uronic acid content. About 0.95 g of sodium tetraborate decahydrates was mixed in 3.0 mL of hot water, after which 97 mL of ice-cold sulfuric acid was poured. The mixture was mixed and cooled. About 0.05 mL of carbazole was pipetted, mixed and heated at 100 °C for 15 min. The mixture was then cooled at room temperature and read at 525 nm. D-Gluconoric acid was used to prepare a standard curve [30].

#### 2.5.2. Surface Morphology and Elemental Composition

A scanning electron microscopy (SEM) (SEM-Sipma-VP03-67, Zeiss and P-Sigma, Carl-Zeiss-Strasse 22, 73447 Oberkochen Germany), equipped with an elemental analyzer, was employed in order to ascertain the bioflocculants surface morphology and elemental composition. The bioflocculant (0.5 mg) was placed on a silicon-coated slide fixed using a spin coater at 1000 rpm for 1 min. Thereafter, the bioflocculant was placed into the instrument, and the surface images of the bioflocculant and elemental composition were captured [31].

### 2.6. Flocculation Characteristics of the Bioflocculant

#### 2.6.1. Effect of Dosage Concentration on Flocculating Activity

The method described by Makapela et al. [32] was utilized to determine the purified bioflocculant dosage concentration effect on flocculating activity. Concentration ranges (0.2–1.0 mg/mL (*w*/*v*)) of the purified bioflocculant were prepared. The bioflocculant solution (2 mL) for each concentration was mixed with the kaolin clay suspension (100 mL, 4 g/L) and 3 mL of 1% (*w*/*v*) CaCl_2_ in a conical flask (250 mL) and vigorously shaken. The mixtures (100 mL) were poured into graduated measuring cylinders (100 mL) and were allowed to stand for 5 min at room temperature to sediment. Thereafter, the flocculating activity of the bioflocculant was determined by withdrawing the supernatant and reading the optical density at 550 nm using a spectrophotometer (Unic-7230, Shanghai Lianhua Company, Shanghai, China). The following equation was used to measure the percentage (%) flocculating activity of the bioflocculant;

$$\mathrm{Flocculating}\;\mathrm{activity}\;(\mathrm{FA})=[\frac{A-B}{A}]\;\times \;100,\;[1]$$

where *A* is the OD at 550 nm of kaolin clay suspension and *B* is the optical density at 550 nm of the treated solution.

#### 2.6.2. Effect of Cations on Flocculating Activity

The method described by Okaiyeto et al. [31] was followed to evaluate the impact of the cations on flocculating activity of the bioflocculant. About 3 mL of 1% CaCl_2_ (*w*/*v*) solution in the standard method, was substituted by dissimilar metal ions such as; Li, K, Na, BaCl_2_, MnCl_2_, FeCl_3_ and AlCl_3_. The mixture of kaolin solution and bioflocculant without any cation served as a control. The flocculating activity was then measured using kaolin solution as described previously (see Section 2.6.1). 

#### 2.6.3. Effect of AlCl_3_ Concentration on Flocculation

The preferable cation (AlCl_3_) for the bioflocculant to achieve the highest flocculating efficiency during the determination of the effect of cation on flocculation was subjected for assessment of the optimum concentration to be utilized. Different concentrations (0.25–1.5 g/mL) of AlCl_3_ were prepared and used prior to the evaluation of the flocculating activity against kaolin solution (4 g/L) [33].

#### 2.6.4. Antibacterial Activity of the Bioflocculant 

The antibacterial activity of the fungal bioflocculant was investigated in terms of the minimum inhibitory concentration (MIC) by a rapid Mueller–Hinton broth micro-dilution method with p-iodonitrotetrazolium violet solution (0.2 mg/mL) as an indicator [34]. Prior to the assessment of MIC, *Staphylococcus aureus* (ATCC 25925) and *Escherichia coli* (ATCC 25922) at exponential growth were adjusted to 1 × 108 colony forming units per millimeters (CFU/mL). Dimethyl sulphoxide (DMSO) (10%) was used as a negative control, while ciprofloxacin served as a positive control [35].

### 2.7. Application of the Bioflocculant in Wastewater Treatment

Wastewater samples were collected from KwaDlangezwa Wastewater Treatment Plant and Tendele Coal Mine Wastewater Plant in KwaZulu-Natal, South Africa. Parameters such as total nitrogen (N), biochemical oxygen demand (BOD), phosphorus (P) and chemical oxygen demand (COD) were measured before and after treatment with the bioflocculant. The measurements were performed using a spectrophotometer (Spectro-quant Phero 300, Merck KGaA, Darmstadt, Germany) and a pH meter (Eutech Instruments pH 700, Singapore) [36]. About 3 mL of 1% (*w*/*v*) AlCl_3_ ions and 2 mL of 0.4 mg/mL bioflocculant solution were mixed with 100 mL of wastewater sample in a 250 mL conical flask. The mixtures were agitated at 200 rpm for 3 min, and the speed was thereafter reduced to 40 rpm for 5 min. The flasks were left at room temperature to stand for 10 min for sedimentation. For comparison, conventional flocculants such as alum and ferric chloride (0.4 mg/mL) were used. Thereafter, the percentage removal efficiency of the bioflocculant on; N, BOD, P and COD was measured by reading the optical densities at 680 nm. The following formula was used to calculate the bioflocculant removal efficiency [37];

$$\mathrm{Removal}\;\mathrm{efficiency}\;(\mathrm{RE})\;(\%)=[\frac{Co-C}{Co}]\;\times \;100,$$

whereby *Co* and *C* are the values before and after the flocculation process measured at 680 nm, respectively [38].

### 2.8. Application of the Bioflocculant in Dye Removal

The dye removal potential of the bioflocculant was evaluated by adopting the method by Pathak et al. [38]. The different dye solutions such as Congo red, nigrosine, methylene blue and safranin (4 g/L) were prepared. In a 100 mL of each dye solution, 2 mL of 0.4 mg/mL bioflocculant solution and 3 mL of 1% (*w*/*v*) AlCl_3_ solution were added. The mixtures were shaken at 200 rpm for 3 min, and the speed was then reduced to 40 rpm for 5 min. The solution without the bioflocculant served as the control. The dye removal efficiency of the bioflocculant was then calculated as described previously.

### 2.9. Statistical Analysis

All data were collected in triplicates with mean and standard deviation values determined where differences were considered significant at 0.05 at confidence level (*p* > 0.05) by the use of Graph Pad Prism version 6. The significance was evaluated by variance analysis (ANOVA).

## 3. Results and Discussions

### 3.1. Extraction and Purification

There is a constant search for bioflocculants characterized by high efficiency and low costs due to the shortcomings of low yields, and high costs present significant practical application constraints in the production of bioflocculants. In the current study, the bioflocculant was extracted from *P. kudriavzevii* MH545928.1. The crude, unpurified bioflocculant obtained was 3.6 g/L. After a partial purification by using a mixture of chloroform and butanol (5:2 *v*/*v*), the fungus gave a yield of 2.836 g/L. The produced bioflocculant by *P. kudriavzevii* MH545928.1 was high compared to the SMP-P bioflocculant yield of 0.172 g/L produced by *Phanerochaete chrysosporium* [39]. However, the yield was much lower when compared to other studies. At a culture duration of 168 h, the maximum biomass accumulation of 9.53 g/L was recorded for bioflocculant produced by *Aspergillus oryzae* [40]. Another high yield (4.52 g/L) was observed from the study of He et al. [41] produced by *Halomonas* sp. V3a, and Wang et al. [42] also reported a yield of 3.8 g/L produced by *Ochrabactium cicero* W2. Studies show that a method of extraction plays a huge role in obtaining a high yield of bioflocculant, as some methods are more preferred than others [43]. Thus, the difference in the yields might not only be attributed to the different abilities of each strain to produce the bioflocculant but also the method employed during extraction and purification processes. 

### 3.2. Solubility of the Bioflocculant

Table 1 shows the results for the solubility assay of the bioflocculant. The bioflocculant was insoluble in all the tested organic solvents but dissolved in water. In our previous study, the bioflocculant was revealed to have the hydroxyl group [44]. Thus, the bioflocculant has the potential to bind one or more water molecules. The presence of hydroxyl groups creates significant interaction between the bioflocculant molecules, leading to a crystalline structure that is rather rigid. Organic solvents cannot break these forces; hence, the purified bioflocculant does not dissolve in any of the tested organic solvents [45].

### 3.3. Chemical Composition of the Bioflocculant

The components of the bioflocculants ought to be identified in order to comprehend their flocculation mechanisms [46]. This would aid in the enhancement of the flocculating parameters, resulting in an increase in the performance during applications. In this study, the chemical analysis of the bioflocculant from *P. kudriavzevii* MH545928.1 was conducted to determine the composition of carbohydrates, proteins and uronic acid. The bioflocculant was found to have a total carbohydrates content of 69%, protein content of 11% and uronic acid content of 16% (Table 2). The results indicate that the bioflocculant is predominately a carbohydrate molecule. This implies that carbohydrates are the most active components during the flocculation process. Moreover, the high carbohydrate content affirmed our previous study that the bioflocculant is thermally stable [24]. Bioflocculants dominated by protein content are known to be heat sensitive as proteins denature easily at high temperatures [47]. Our findings are in agreement with observations found in other studies. The total carbohydrate content of 23 mg/mL was reported by Mabinya et al. [48], with proteins absent from the bioflocculant. Xiong et al. [49] also reported a bioflocculant with carbohydrates (89%) and protein contents (11%). 

### 3.4. Surface Morphology of the Purified Bioflocculant

**Figure 1:**
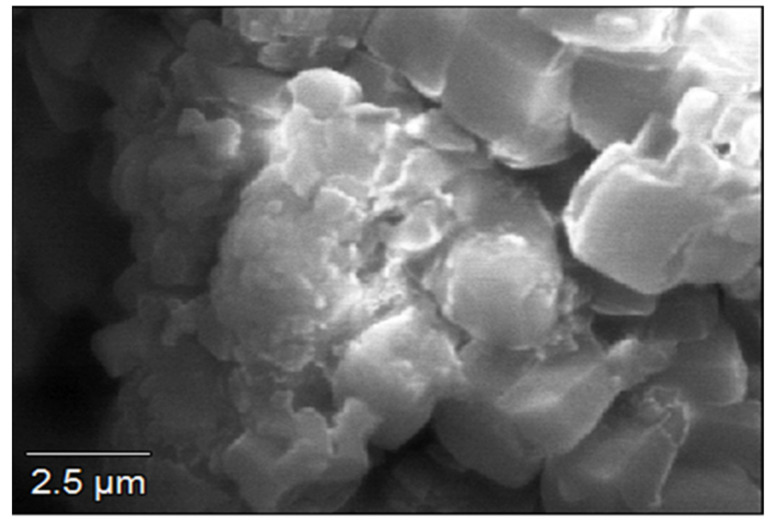
Surface morphology of the bioflocculant.

In the flocculation process, the surface morphological structure of the bioflocculant is of great importance [49]. It determines whether bioflocculants would be effective or ineffective during the flocculation process. The bioflocculant displayed a cumulus-like structure under SEM (Figure 1). The bioflocculant’s high flocculating activity was, therefore, attributed to its configuration. Nie et al. [40] observed the surface morphology of the bioflocculant produced by *Aspergillus oryzae* to be porous, fibrous and wrinkled. The surface areas of the fungal particles were enhanced due to the filamentary spatial arrangement. The SEM surface morphology of the bioflocculant p-KG03 had a fibrous type of structure [50]. The bioflocculant from *P. chrysosporium* showed cotton-like nanofibers [51]. This finding demonstrated that fungal pellets have different configurations when viewed from SEM, and these surface morphologies enable individual bioflocculants to be effective during their applications.

### 3.5. Elemental Analysis of the Bioflocculant

**Figure 2:**
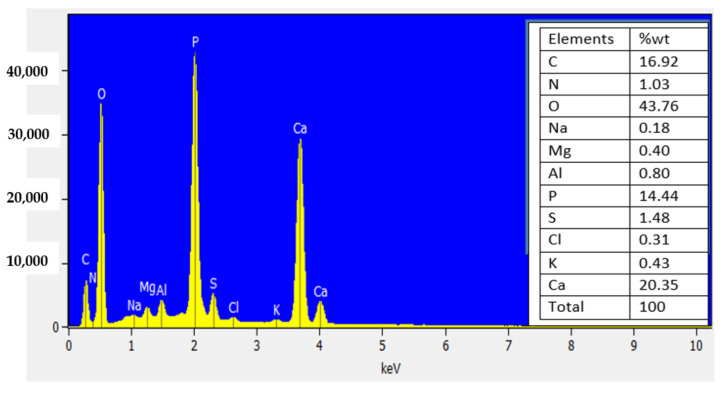
Elemental analysis of the bioflocculant.

Bioflocculant structure and flocculating activity are influenced by the elemental composition of the bioflocculants. The bioflocculants’ flexibility and stability are aided by various elements [52]. The adsorption peaks are shown by the elemental spectrum, which indicates the presence of elements such as C, N, O, Na, Mg, Al, P, S, Cl, K and Ca, which accounts for 16.92: 1.03: 43.76: 0.18: 0.40: 0.80: 14.44: 1.48: 0.31: 0.34: 20.35 (% wt), respectively (Figure 2). The presence of C, N and O in the bioflocculant affirms that the bioflocculant in this study is a glycoprotein polymer (see Table 1). Moreover, the bioflocculant presence of these elements might contribute effectively to its flocculation capability. The observed results were similar to those of the bioflocculant produced by *Halobacillus* sp. [52]. The bioflocculant’s elemental analysis reported the following percentages: 42.03: 5.31: 21.44: 5.83: 0.85 (% wt) for C, N, O, P and S, respectively. Moreover, the study by Lu et al. [53] reported that a bioflocculant produced by *Enterobacter aerogenes* had elements such as C: O: N: S: with 39.8: 52.6: 0.8: 0.2 (% wt), respectively.

### 3.6. Dosage Concentration of the Bioflocculant

In the flocculation process, dosage concentration is a critical factor [54]. Inadequate or excessive dosage can lead to inhibition or a decrease in flocculation. Furthermore, an appropriate dose size helps to minimize expenses and increase flocculation efficiency [55]. Thus, the effect of the bioflocculant dosage on flocculating efficiency was studied, and the results are demonstrated in Table 3. The dosage concentration of 0.4 mg/mL was used for all the experiments that followed as it showed the best flocculating activity of 80.01%. However, the flocculation rate was poor at a lower concentration (0.2 mg/mL), which could be due to the negative charges on kaolin particles that were not neutralized due to inadequate bioflocculant concentration. At higher concentrations (0.4 mg/mL < x), the charges were assumed to have been excessive, leading to a reduction in the flocculation rate [56]. Deng et al. [57] reported that higher or lower doses were also shown to cause lower performance on a bioflocculant. 

### 3.7. Effect of Cations on the Bioflocculant

The effect of cations on flocculating activity of the purified bioflocculant was investigated, and the results are shown in Table 4. The flocculating activity of the bioflocculant produced by *P. kudraivzevii* MH545928.1 was highly enhanced by the addition of Al^3+^ as a trivalent cation. Other tested cations showed a lesser effect on the flocculating activity. The lowest flocculation rate was in the presence of Ba^2+^. Thus, Al^3+^ stimulated flocculation through destabilization and neutralization of residual negative charges of the functional groups of the bioflocculant and kaolin particles, resulting in the bridge formations that bound the kaolin particles together [58]. However, one study documented that trivalent cations such as Fe^3+^ tend to inhibit flocculation [59]. The bioflocculant p-KG03 produced by *Gyrodinium impudicum* KG03 was not enhanced by the addition of any cations, which indicates that some bioflocculants are cation-independent [50]. Al^3+^ was used in the following experiments in this study.

### 3.8. Effect of AlC_3_ Concentration on the Bioflocculant

It is important to determine the optimum cation concentration to best flocculant particles as this helps to reduce costs while maximizing activity. The effect of different concentrations of AlCl_3_ on the flocculating activity of the bioflocculant was evaluated. The bioflocculant flocculating activity was very positive for all the concentrations, with the highest activity of 80% obtained at 1.25 g/L (Table 5). Thus, 1% of Al^3+^ was optimum to stimulate flocculation through destabilization and neutralization of negative charges of the bioflocculant and kaolin particles. Zhang et al. [60] reported similar findings with bioflocculant from *Mycobacterium nannocystis* sp. NU-2. The flocculation activity of the bioflocculant increased with an increase in AlCl_3_ concentration up to 99% flocculation activity at 30 mg/L.

### 3.9. Antibacterial Effect of the Bioflocculant

Bioflocculants with antimicrobial activities are good natural disinfectants for use in wastewater treatment [61]. Table 6 shows the MIC of the bioflocculant. It exhibited some notable antibacterial properties against both Gram-positive and Gram-negative bacteria. It gave the MIC values of 3.125 mg/mL against *E. coli* (ATCC 25922) and 1.563 mg/mL on *S. aureus* (ATCC 25925) (Table 3). *S. aureus* (ATCC 25925) was more susceptible in comparison to *E. coli* (ATCC 25922), which showed more resistance. Gram-negative bacteria, in comparison to the Gram-positive strains, are generally resistant to most antibacterial agents due to their outer membrane, which tends to prevent some antibacterial agents from penetrating the bacteria and exerting their action. Nevertheless, the activity was not noteworthy (1 < MIC) against both used bacteria. Similar findings were reported by Giri et al. [61], where bioflocculant PB showed un-noteworthy inhibition against the tested bacterial strains. However, to some extent, the bioflocculant might have the potential to be used for dual purposes, which is for flocculation and disinfection. This can, thus, be cost-effective.

### 3.10. Application of the Purified Bioflocculant

#### 3.10.1. Wastewater Treatment

The bioflocculant produced by *P. kudriavzevii* MH545928.1 was used to remove impurities such as COD, BOD, N and P in domestic wastewater. The bioflocculant showed the removal efficiencies of 73, 49, 47 and 50% for BOD, COD, P and N, respectively (Table 4). It is worth noting that the bioflocculant revealed the highest removal efficiencies than the conventional flocculants teste, FeCl_3_ (27, 36, 46 and 40% for BOD, COD, P and N, respectively and alum (47, 33, 29 and 40% for BOD, COD, P and N, respectively) in all parameters (Table 7). The effectiveness of wastewater treatment is mostly conveyed in terms of reduction in the chemical oxygen demand (COD) and biological oxygen demand (BOD). The high levels of COD and BOD in wastewaters do not sustain aquatic life and result in foul odors and anaerobic conditions, which consequently lead to death [62]. Moreover, although N and P are essential nutrients for plant growth, the excessive deposition of them in terrestrial or oceanic ecosystems may result in eutrophication and water pollution [44]. Thus, it is of utmost importance to remove excess N and P species from wastewater before it is discharged into different water bodies. Similar results were observed by Gong et al. [56], whereby the bioflocculant SF-1 demonstrated higher removal efficiencies in comparison to the conventional flocculants. Moreover, the findings are also comparable with the results from Dlamini et al. [63], where the bioflocculant from *Alcaligenes faecalis* had profound removal efficiencies for N and P.

The bioflocculant was also evaluated for its removal efficiency on coal mine wastewater, and the results are illustrated in Table 8. About 3 mL of 1% (*w*/*v*) AlCl_3_ ions and 2 mL of 0.4 mg/mL bioflocculant solution were mixed with 100 mL of wastewater sample in a 250 mL conical flask. The bioflocculant had better removal efficiencies for BOD (64%) compared to the conventional flocculants. The removal efficiencies on COD and BOD were 78% and 75%, respectively, while for FeCl_3_ were 71% and 72%, respectively. Kaur et al. [64] presented a bioflocculant capable of removing the impurities from raw composting leachate such as phosphorus and COD, with removal rates of 92% and 69%. The produced bioflocculant was competitive for the removal of impurities from both domestic wastewater and coal mine wastewater, and therefore, the bioflocculant has the potential to be used in treatments of various wastewater, especially industrial and domestic wastewater.

#### 3.10.2. Application of the Bioflocculant from Various Dye Solutions

Two anionic dyes (Congo red and nigrosine) and two cationic dyes (methylene blue and safranin) were used to evaluate the dye removal efficiency of the bioflocculant. The decolorization ability of the bioflocculant varied depending on the dye used. According to the findings depicted in Table 9, both anionic dyes had a removal rate of 81%. The bioflocculant revealed the removal ability of 73% against methylene blue and 74% on safranin. As the availability and strength of positive charges in the solution, which were fixed by the confirmation and cationicity of the bioflocculant, had a direct effect on the removal of anionic dyes [65]. The pH affects the electrochemistry of the dyes and the dissociation of the polyelectrolytes and, thus, the conformation in the solution [66]. Because of the cationic property of the bioflocculant, the effect of pH on dye removal is not apparent for cationic dyes, therefore the low removal activities. Overall, the bioflocculant had the modest capacity to remove anionic dyes (Congo red and nigrosine). These findings indicated that the bioflocculant might be more effective in the removal of anionic dyes than cationic dyes. Chen et al. [67] also reported in a bioflocculant produced by *Alteromonas* sp. CGMCC 10612 with the highest removal rate for the anionic dyes, Congo red and direct black, with a removal rate of 98.5% and 97.9%, respectively. In another study, a bioflocculant produced from *Bacillus megaterium* was able to remove methylene blue with a removal efficiency of 64.9% [68]. In conclusion, the bioflocculant produced by *P. kudriavzevii* MH545928.1 can be used in various dye-producing textile wastewater industries for the removal of different wastewater dyes.

## 4. Conclusions

Bioflocculants are thought to have great industrial and biotechnological significance. *P. kudriavzevii* MH545928.1 produced a bioflocculant of 2.836 g/L after extraction and purification, which was better compared to some of the studies. The bioflocculant was insoluble to all the tested organic solvents but soluble in water. It is a glycoprotein molecule composed of carbohydrates, proteins and uronic acid. The elemental analysis affirmed that this bioflocculant was a glycoprotein polymer. It demonstrated moderate antibacterial activity, implying its potential to be used as a flocculant and disinfectant. The bioflocculant was relatively effective in dye removal and wastewater treatment when compared to conventional flocculants. For further studies, more characterization of the bioflocculant and determination of the flocculation mechanisms are imperial.

## Figures and Tables

**Table 1:** Solubility of the purified bioflocculant.

Solvents	Solubility
Water	+
Chloroform	−
Ethanol	−
Methanol	−
Butanol	−

Keys: + denotes dissolve and − signify undissolved.

**Table 2:** Chemical compositions of the purified bioflocculant.

Samples	Percentage (%)
Carbohydrates	69
Uronic acids	16
Proteins	11

**Table 3:** Effect of dosage on flocculation.

Dosage (mg/mL)	FA (%) ± SD
0.2	54.03 ± 2.02 ^a^
0.4	80.01 ± 1.05 ^b^
0.6	59.00 ± 4.01 ^a^
0.8	51.21 ± 6.07 ^a^
1.0	28.04 ± 11.02 ^ab^

Different letters (a, b) denotes statistical significance at (*p* < 0.05).

**Table 4:** Effect of cations on flocculation.

Cations	FA (%) ± SD
Na	54.61 ± 2.03 ^a^
Li	44.03 ± 11.31 ^a^
K	52.31 ± 7.20 ^a^
Ba^2+^	35.00 ± 1.31 ^a^
Mn^2+^	61.01 ± 21 ^ab^
Fe^3+^	50.00 ± 1.10 ^a^
Al^3+^	72.24 ± 0.35 ^ab^
Control	16.33 ± 5.35 ^c^

Different letters (a, b, c) denotes statistical significance at (*p* < 0.05).

**Table 5:** Effect of AlCl_3_ concentrations on flocculation.

AlCl_3_ (g/L)	FA (%) ± SD
0.25	71.00 ± 1.25 ^a^
0.5	72.14 ± 3.43 ^a^
0.75	75.32 ± 6.20 ^a^
1.0	72.30 ± 0.04 ^a^
1.25	80.1 ± 3.05 ^ab^
1.5	61.22 ± 1.04 ^b^

Different letters (a, b) denotes statistical significance at (*p* < 0.05).

**Table 6:** MIC values of the bioflocculant against the test bacteria.

Bacterial Strains	Bioflocculant (mg/mL)	Ciprofloxacin (µg/mL)
*E. coli* (ATCC 25922)	3.125	0.015
*S. aureus* (ATCC 25925)	1.563	0.015

**Table 7:** The removal efficiency of the bioflocculant on domestic wastewater.

Types of Flocculants		Removal Efficiency (%)			
	Water Quality	BOD	COD	P	N
Bioflocculant	Before treatment	1.5	320	7	10
	After treatment	0.4	162	4	5
	Removal effeciency	73 ^a^	49 ^b^	47 ^b^	50 ^b^
Alum	Before treatment	1.5	320	7	10
	After treatment	0.8	214	5	6
	Removal efficiency	47 ^b^	33 ^d^	29 ^d^	40 ^c^
FeCl_3_	Before treatment	1.5	320	10	10
	After treatment	1.1	204	6	6
	Removal efficiency	27 ^d^	36 ^c^	46 ^b^	40 ^c^

Different letters (a, b, c and d) denotes statistical significance at (*p* < 0.05).

**Table 8:** The removal efficiency of the bioflocculant on coal mine wastewater.

Types of Flocculants		Removal Efficiency (%)			
	Water Quality	BOD	COD	P	N
Bioflocculant	Before treatment	4.2	435	8	8
	After treatment	1.5	247	5	4
	Removal effeciency	64 ^a^	43 ^c^	38 ^c^	50 ^b^
Alum	Before treatment	4.2	435	8	8
	After treatment	2.7	249	3	4
	Removal efficiency	36 ^c^	40 ^c^	63 ^a^	50 ^b^
FeCl_3_	Before treatment	4.2	435	8	8
	After treatment	1.8	205	4	5
	Removal efficiency	57 ^ab^	53 ^bc^	50 ^b^	38 ^c^

Different letters (a, b, c) denotes statistical significance at (*p* < 0.05).

**Table 9:** Dye removal efficiencies of the bioflocculant from *P. kudriavzevii* MH545928.1.

Dyes	Bioflocculant FA (%) ± SD	FeCl_3_ FA (%) ± SD	Alum FA (%) ± SD
Congo red	81.03 ± 1.04 ^a^	72.23 ± 3.06 ^a^	81.05 ± 0.22 ^a^
Nigrosine	81.20 ± 0.13 ^a^	84.01 ± 1.44 ^a^	85.00 ± 3.23 ^a^
Methylene blue	73.41 ± 2.11 ^a^	65.09 ± 0.32 ^b^	87.21 ± 0.41 ^a^
Safranin	74.35 ± 3.05 ^a^	65.11 ± 0.45 ^b^	79.06 ± 1.05 ^a^

Different letters (a, b) denotes statistical significance at (*p* < 0.05).

## Data Availability

Not applicable.
